# T-L Plane Abstraction-Based Energy-Efficient Real-Time Scheduling for Multi-Core Wireless Sensors

**DOI:** 10.3390/s16071054

**Published:** 2016-07-08

**Authors:** Youngmin Kim, Ki-Seong Lee, Ngoc-Son Pham, Sun-Ro Lee, Chan-Gun Lee

**Affiliations:** 1 Department of Computer Science and Engineering, Chung-Ang University, Heuksuk-ro 84, Dongjak-gu, Seoul 156-756, Korea; remnant1120@gmail.com (Y.K.); goory00@gmail.com (K.-S.L.); ssunno@cau.ac.kr (S.-R.L.); 2 School of Electrical and Electronics Engineering, Chung-Ang University, Heuksuk-ro 84, Dongjak-gu, Seoul 156-756, Korea; phamngocson1408@gmail.com

**Keywords:** energy efficiency, wireless sensor node, T-L plane, real-time scheduling, DPM

## Abstract

Energy efficiency is considered as a critical requirement for wireless sensor networks. As more wireless sensor nodes are equipped with multi-cores, there are emerging needs for energy-efficient real-time scheduling algorithms. The T-L plane-based scheme is known to be an optimal global scheduling technique for periodic real-time tasks on multi-cores. Unfortunately, there has been a scarcity of studies on extending T-L plane-based scheduling algorithms to exploit energy-saving techniques. In this paper, we propose a new T-L plane-based algorithm enabling energy-efficient real-time scheduling on multi-core sensor nodes with dynamic power management (DPM). Our approach addresses the overhead of processor mode transitions and reduces fragmentations of the idle time, which are inherent in T-L plane-based algorithms. Our experimental results show the effectiveness of the proposed algorithm compared to other energy-aware scheduling methods on T-L plane abstraction.

## 1. Introduction

A wireless sensor network (WSN) consists of spatially-distributed autonomous sensors to measure/monitor various conditions and transmit the collected data using wireless communications. WSNs are considered as a promising approach, enabling a wide spectrum of applications, such as area surveillance, traffic flow measurement, object tracking, and environment monitoring.

Due to the emerging demands for advanced applications, such as video sensor networks with image sensors and smart cameras, single-core embedded wireless sensor nodes face high-performance computation challenges. Recent technological improvements rendered multi-core processors as a viable and cost-effective option for coping with the computation challenges for sensor nodes [1]. Hence, studies on multi-core sensor nodes have been actively conducted recently [2,3,4].

Such multi-core sensor nodes require energy-efficient real-time scheduling algorithms to meet their timing requirements, accomplished by exploiting the multi-core processors, and to keep the battery life long enough to achieve such a goal. Among the real-time scheduling algorithms, the T-L plane-based scheme is known to be an optimal global scheduling technique for periodic real-time tasks on multi-cores. Unfortunately, there has been a scarcity of studies on extending T-L plane-based scheduling algorithms to exploit energy-saving techniques.

Voltage frequency scaling (VFS) [5] and dynamic power management (DPM) [6] are the most frequently adopted techniques for saving dynamic power dissipation. VFS scales the voltage and frequency of a processor in order to reduce the energy consumption. DPM exploits the idle time of a processor and switches the processor to lower energy consumption modes, such as sleep or deep sleep modes.

In this paper, we propose new events and associated algorithms to enable better energy management for multi-core sensor nodes operating with a T-L plane-based scheduling algorithm. More specifically, we extend the approach made previously [7] by considering the characteristics of multi-core sensor nodes as follows.

- Generalization of the technique for prefetching the tokens originally scheduled to later planes; and
- Allocation of the minimum number of preprocessors at the beginning of each T-L plane.

The first extension is especially important for the T-L plane algorithm, which can frequently generate a series of short idle blocks, as shown in Section 2. The previous technique [7] was generalized to prefetch the tokens originally scheduled to the future planes, as well as the very next one, and then execute them during an idle interval whose length is too short to switch the processor into sleep mode. These efforts reduce the fragmented idle time durations and increase the chance of longer idle blocks where the sensor processors can be placed into sleep mode.

The second extension is pursued due to the observation of work load distributions in some sensor network applications. Note that the load of the task sets may significantly change in different time frames, such as during the daytime and nighttime. An imbalance of load indicates that there is an opportunity of turning some processors off during lighter load times. For example, a recent study [8] on load monitoring stated that their system runs monitoring tasks heavily around midnight when the frogs are active. However, the system experiences silence during the daytime. Another example is energy-harvesting sensor networks, where day and night tasks must be different due to the availability of sunlight. There is an abundance of similar situations in various sensor networks. The idea is similar to the one used by chilled water plant engineers to solve chiller dispatching problems where optimization algorithms decide when to turn the chillers on and off [9].

This paper is organized as follows: Section 2 introduces major previous work involving real-time scheduling and energy management. In Section 3, a short review of T-L plane abstraction is presented. In Section 4, we present new events and associated algorithms extending the T-L plane scheduling to support the dynamic power management (DPM) technique. In addition, some theoretical findings are discussed. In Section 5, we evaluate our algorithm by comparing it with other T-L plane-based energy-efficient algorithms. In Section 6, we summarize the results of this study and suggest future work.

## 2. Related Work

This section briefly introduces the topic of multi-processor scheduling. It also summarizes previous major works on T-L plane-based real-time scheduling algorithms and associated extension efforts toward energy-efficiency. Interested readers are referred to extensive surveys of energy-efficient scheduling mechanisms on sensor networks [10] and energy-aware real-time scheduling algorithms [11].

Research on real-time scheduling for multi-processors has largely been focused on the problem of scheduling periodic tasks. The real-time scheduling algorithms on multi-processors for periodic tasks are categorized into global or partitioning-based scheduling [12,13,14]. In global scheduling [15,16], task migrations between the processors are allowed since all of the tasks waiting for execution are in a single queue, where a task scheduler picks some tasks. In contrast, task migration is not permitted in partition-based scheduling. Each processor has its own waiting queue and an independent task scheduler.

The T-L plane-based scheme is an optimal global scheduling for independent real-time tasks on a homogeneous multi-processor system [17]; there has been active research on the extension of this scheme since its seminal paper was published. A study involving synchronization mechanisms for lock-based, lock-free, and wait-free schemes in largest nodal remaining execution-time first (LNREF) scheduling was previously presented [18]. The extension of T-L plane scheduling to support sporadic tasks was also performed [19]. An optimal work-conserving scheduling was proposed to reduce the idle time in LNREF scheduling [20,21]. In addition, an approach to reduce task migrations in a T-L plane was presented [22,23].

Recent advances in T-L plane-based scheduling have started to consider energy efficiency. Most approaches propose to determine the frequencies and voltages of the processors rendering minimal energy consumption [24,25,26,27]. According to supply voltages and operation frequencies in a CMOS processor, the dynamic power consumption for charging and discharging switching capacity $P_{d}$ can be computed as shown in the following:

(1) $$P_{d}=\alpha CV^{2}f$$

where α is switching activity factor, C is switching capacitance, V is supply voltage, and f is frequency. The VFS technique reduces dynamic power consumption by scaling supply voltage and operation frequency of processors. It also reduces the power consumed by short circuit current appearing at rise and fall time of input signal. In contrast, DPM turns out more effective than VFS when there is enough idle time because DPM enables the shutdown of processors and decrease of supply voltage. DPM can reduce the static power consumed by leakage current, too.

Unfortunately, there has been little effort to extend T-L plane-based scheduling algorithms to support the DPM technique, despite the popularity of multi-processors and the issue of increasing dynamic power. Zhang et al. [27] briefly mentioned the possibility of switching the processor’s mode to utilize idle time. However, it focuses exclusively on DVFS. We argue that such a basic attempt to apply the idea may not work, as shown in the following example.

**Figure 1:**
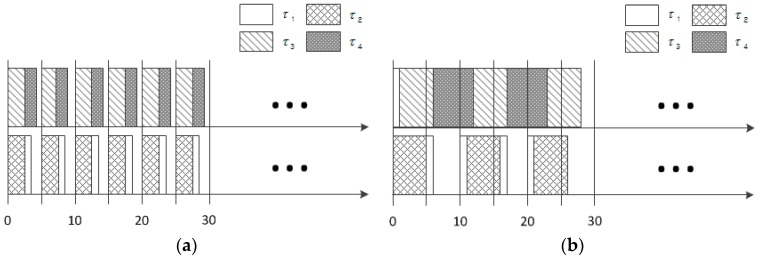
Example of idle time fragmentation: (**a**) LNREF schedule and (**b**) global-EDF schedule.

Figure 1 shows the schedules produced by LNREF and global-EDF [14,28] on two processors. It is noticeable that a series of idle times appear frequently in the schedule by LNREF compared to the schedule generated by global earliest deadline first (global-EDF). More specifically, there are two idle blocks with durations of 1 ms and 2 ms on every plane of the two processors, as shown in Figure 1a. This is primarily due to the characteristics of T-L plane-based scheduling, where a task is broken down into a token of each plane whose deadline is the end time of the plane. The reason why we care about this issue is that such short durations of the idle time may not be long enough to exploit more energy-efficient modes, such as sleep mode.

TL-DPM [7] recently addressed this issue and proposed the idea of executing ahead of time the tasks originally scheduled for the plane that immediately follows when the idle time duration is too short to switch to sleep mode. This approach is based on the rationale that such actions can contribute to reduce the fragmented idle time durations and render them into larger blocks.

## 3. Review of T-L Plane Abstraction

In this section, we briefly review the concept of the T-L plane abstraction. Some scheduling algorithms on multi-processors adopt the concept of fluid schedule to achieve optimality. The core idea behind fluid schedule is to execute each task at a constant rate. Such scheduling algorithms frequently switch the context to satisfy the fluid schedule. Cho et al. [17] proposed T-L plane abstraction to address this problem. In the T-L plane abstraction, a task is represented as a moving token. The x-axis and y-axis represent time and tasks’ remaining execution time, respectively.

Figure 2 shows an example of T-L plane construction. For the *jth* job of a task $\tau_{i}$ with arrival at $a_{i,j}$ and cost $C_{i}$, it should be executed and meet its implicit deadline before the arrival of the next job. Each arrival of a job is indicated by a down-directed arrow, which is extended by a dotted vertical line in the figure. The dotted slopes from $\left(a_{i,j},C_{i}\right)$ to $\left(0,a_{i,j+1}\right)$ represent the fluid schedule of tasks. Note that there are the same *n* isosceles triangles for *n* tasks given a pair of consecutive dotted vertical lines, e.g., the ones extended from $a_{3,5}$ and $a_{2,3}$. The height of each isosceles triangle is same to the interval length of the pair. Hence, the rightmost vertex of each isosceles triangle is an intersection point of its fluid schedule and a dotted vertical line. Then, we overlap these triangles in the same time intervals to construct a T-L plane. Figure 2 shows examples of constructing the *k-1th*, *kth*, and *k+1th* T-L planes.

Figure 3 illustrates scheduling in the *kth* T-L plane. A token represents the status of the corresponding task in the plane. Throughout this paper, the start time and finish time of the *kth* plane are represented as $t_{0}^{k}$ and $t_{f}^{k}$, respectively. The occurrence time of each event on the *kth* plane is denoted as $t_{j}^{k}$, where 0≤j≤f. We shall use a simpler notation $t_{j}$ to indicate the occurrence time of an event in the current plane. At time $t_{0}^{k}$, a token corresponding to the task $\tau_{i}$ is located on the left-most side of the *kth* T-L plane, and its height represents the local remaining execution time $l_{i}^{k}\left(t_{0}^{k}\right)$. Assume that we have *m* processors. The tokens of tasks assigned to the processors move diagonally down as $\tau_{1}$,…, $\tau_{m}\;$, or else horizontally as $\tau_{m+1}$,…, $\tau_{n}$ at $t_{0}^{k}$, as shown in Figure 3.

In general, there are two time instants where the system has to reschedule tasks. One instant is when a token hits the “zero local remaining execution time bottom”, which means that the local remaining execution time of the task is completely consumed. The processor that has executed the task now becomes available to run another ready task with the highest local utilization. We refer to this as event-b. An example is shown at time $t_{b}^{k}$ in the figure. In the *kth* T-L plane, the local utilization $r_{i}^{k}\left(t\right)$ for a task $\tau_{i}$ at time t can be calculated by the expression $l_{i}^{k}\left(t\right)/t_{f}^{k}-t$, which amounts to the processor capacity needed by the task. The other instant is when a token hits the “no local laxity diagonal”, which means that the local laxity of the task becomes zero and the corresponding task must be executed immediately. Such an event is referred to as event-c. Two examples are shown in the figure at times $t_{c}^{k}$ and $t_{c+1}^{k}$. Note that successful arrival of all of the tokens at the right apex means that the corresponding task set is locally feasible.

## 4. Extending T-L Plane-Based Scheduling Algorithms to Exploit DPM

In general, a DPM-enabled processor provides three different operating modes: active, idle, and sleep. The sleep mode consumes less energy than any other mode. In order to run a task, the processor should be in the active mode. However, it consumes the largest amount of energy. A basic strategy is to keep the processor in sleep mode as much as possible. Note that switching a processor mode requires additional energy. Hence, it would be wise to switch the processor to sleep mode only if the idle interval is longer than a threshold. Such a threshold is called the break-even time [29] and is denoted as $C_{sleep}$ in this paper. $C_{sleep}$ is defined as follows:

(2) $$C_{sleep}=\max\left(t_{sw},\frac{E_{sw}-P_{sleep}\cdot t_{sw}}{P_{idle}-P_{sleep}}\right)$$

where $E_{sw}$ and $t_{sw}$ denote transition energy overhead and recovery time, respectively. The idle power and sleep power are denoted as $P_{idle}$ and $P_{sleep}$, respectively.

Next, we describe our idea to extend T-L plane-based scheduling algorithms to exploit Dynamic Power Management (DPM).

### 4.1. Processor Mode Transition Strategy

#### 4.1.1. Mode Transition at the Beginning of the Plane

Typical T-L plane-based scheduling algorithms try to utilize all of the available processors at the beginning of each T-L plane. We argue that this decision may negatively affect the energy efficiency. For example, suppose we have five processors and the total local utilization is 1.5 for a specific T-L plane that has six tasks. Then, the original T-L plane-based algorithm assigns the tasks to all of the five processors. Note that the task set is schedulable with only two processors and we could have switched the unused three processors to sleep mode during this plane. Therefore, we propose an algorithm to execute the tasks with the minimum number of processors and keep unnecessary processors in sleep mode as much as possible.

Algorithm 1 presents the logic for determining the minimum number of active processors upon the occurrence of a task arrival event for the *k* plane. This algorithm is executed at the beginning of each plane.
**Algorithm 1** Mode Transition at Task Arrival Event
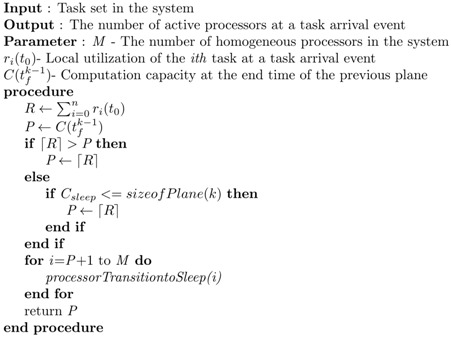


In the algorithm, processorTransitiontoSleep() is a function that switches a processor to sleep mode. Notice that $\left\lceil U_{total}\right\rceil$ processors are enough to schedule the total utilization of $U_{total}$. Hence, the algorithm uses, at most, $\left\lceil U_{total}\right\rceil$ processors and places $M-\lceil U_{total}\rceil$ processors into sleep mode throughout the entire plane.

Hereinafter, the computation capacity at time *x* is notated as C(x), which represents the number of processors in the active mode. For example, $C\left(t_{f}^{k-1}\right)$ indicates the available computation capacity at the end of the previous plane, where the current plane is the *kth* one. If it is smaller than or equal to the minimum number of processors for scheduling $\sum_{i=1}^{n}r_{i}\left(t_{0}\right)$ at $t_{0}$ in the kth plane, which can be computed by $\left\lceil \sum_{i=1}^{n}r_{i}\left(t_{0}\right)\right\rceil$, then *P* is assigned to $\lceil \sum_{i=1}^{n}r_{i}\left(t_{0}\right)\rceil .$ Otherwise, *P* is assigned to $\left\lceil \sum_{i=1}^{n}r_{i}\left(t_{0}\right)\right\rceil$ or $C\left(t_{f}^{k-1}\right)$, depending on the comparison result between the size of the plane and $C_{sleep}$. If the size of the plane is larger than or equal to $C_{sleep}$, then we assign *P* to $\left\lceil \sum_{i=1}^{n}r_{i}\left(t_{0}\right)\right\rceil$ in order to exploit the energy saving of sleep mode.

#### 4.1.2. Mode Transition during Execution

In case the total local utilization decreases as time lapses, the number of processors required for scheduling may be also reduced. Notice that we can switch the unnecessary processors to sleep mode for better energy management. Unfortunately, conventional T-L plane algorithms fail to exploit this opportunity.

In order to address this problem, our approach triggers an event, which is called event-t hereafter, at $t_{t}$ in the case $\lceil \sum_{i=1}^{n}r_{i}\left(t_{t}\right)\rceil <C\left(t_{t-1}\right)$ where $t_{f}-t_{t}>C_{sleep}$. This event implies that the remaining tasks can be scheduled with only $\lceil \sum_{i=1}^{n}r_{i}\left(t_{t}\right)\rceil \;$processors. Hence, the other processors are put into sleep mode.

**Definition 1.**  *An event-t occurs at $t_{t}$ if the following conditions are satisfied*.

- $t_{f}-t_{t}>C_{sleep}$
- $\lceil \sum_{i=1}^{n}r_{i}\left(t_{t}\right)\rceil <C\left(t_{t-1}\right)$

**Figure 4:**
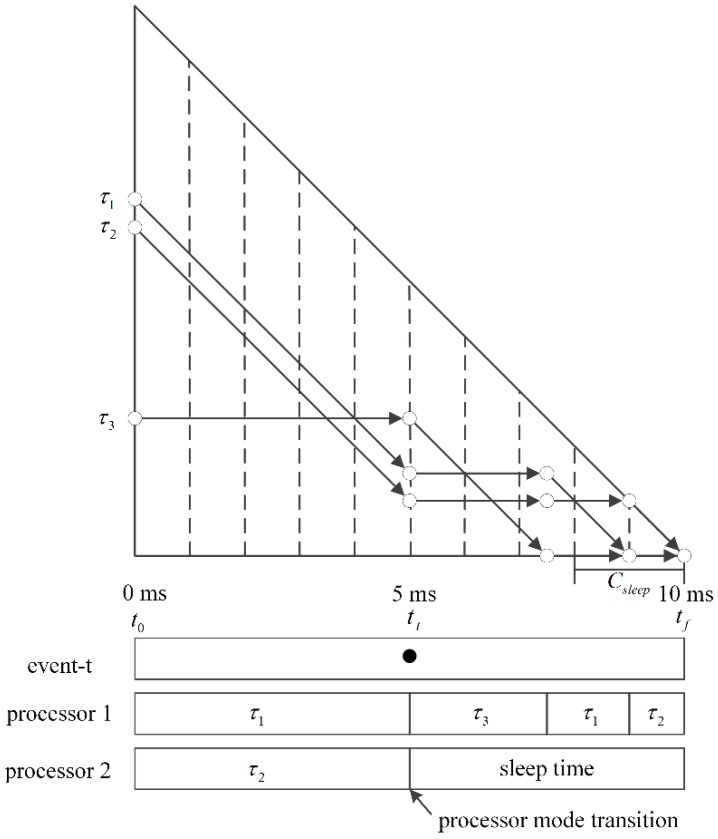
Example of the occurrence of an event-t.

Figure 4 shows an example of the occurrence of event-t. The following theorems and lemmas hold for event-t.

**Theorem 1.**  *In case event-t occurs at $t_{t}$ where $t_{f}-t_{t}>C_{sleep}$, then the following inequalities hold*.

- $\sum_{i=1}^{n}r_{i}\left(t_{t-1}\right)-\lfloor \sum_{i=1}^{n}r_{i}\left(t_{t-1}\right)\rfloor \leq r_{C\left(t_{t-1}\right)}\left(t_{t-1}\right)$
- $1-\left(\sum_{i=1}^{n}r_{i}\left(t_{t-1}\right)-\lfloor \sum_{i=1}^{n}r_{i}\left(t_{t-1}\right)\rfloor \right)\leq r_{C\left(t_{t-1}\right)+1}\left(t_{t-1}\right)$

**Proof:**  The occurrence of even-t at $t_{t}$ requires that $t_{t-1}+\left(\sum_{i=1}^{n}r_{i}\left(t_{t-1}\right)-\lfloor \sum_{i=1}^{n}r_{i}\left(t_{t-1}\right)\rfloor \right)\left(t_{f}-t_{t-1}\right)$ should precede the moment where the local remaining execution time of the $C\left(t_{t-1}\right)$*th* task becomes zero, which is $t_{t-1}+l_{C\left(t_{t-1}\right)}\left(t_{t-1}\right)$.

(3) $$t_{t-1}+\left(\overset{n}{\underset{i=1}{\sum }}r_{i}\left(t_{t-1}\right)-\lfloor \overset{n}{\underset{i=1}{\sum }}r_{i}\left(t_{t-1}\right)\rfloor \right)\left(t_{f}-t_{t-1}\right)\leq \;t_{t-1}+l_{C\left(t_{t-1}\right)}\left(t_{t-1}\right)$$

(4) $$\overset{n}{\underset{i=1}{\sum }}r_{i}\left(t_{t-1}\right)-\lfloor \overset{n}{\underset{i=1}{\sum }}r_{i}\left(t_{t-1}\right)\rfloor \leq \frac{l_{C\left(t_{t-1}\right)}\left(t_{t-1}\right)}{t_{f}-t_{t-1}}$$

(5) $$\overset{n}{\underset{i=1}{\sum }}r_{i}\left(t_{t-1}\right)-\lfloor \overset{n}{\underset{i=1}{\sum }}r_{i}\left(t_{t-1}\right)\rfloor \leq r_{C\left(t_{t-1}\right)}\left(t_{t-1}\right)$$

In addition, it should precede the moment where the local laxity of the $C\left(t_{t-1}\right)$+1*th* task becomes zero, which is $t_{t-1}+\left(t_{f}-t_{t-1}-l_{C\left(t_{t-1}\right)+1}\left(t_{t-1}\right)\right)$.

(6) $$t_{t-1}+\left(\overset{n}{\underset{i=1}{\sum }}r_{i}\left(t_{t-1}\right)-\lfloor \overset{n}{\underset{i=1}{\sum }}r_{i}\left(t_{t-1}\right)\rfloor \right)\left(t_{f}-t_{t-1}\right)\leq \;t_{t-1}+\left(t_{f}-t_{t-1}-l_{C\left(t_{t-1}\right)+1}\left(t_{t-1}\right)\right)$$

(7) $$\overset{n}{\underset{i=1}{\sum }}r_{i}\left(t_{t-1}\right)-\lfloor \overset{n}{\underset{i=1}{\sum }}r_{i}\left(t_{t-1}\right)\rfloor \leq \frac{t_{f}-t_{t-1}-l_{C\left(t_{t-1}\right)+1}\left(t_{t-1}\right)}{t_{f}-t_{t-1}}$$

(8) $$\overset{n}{\underset{i=1}{\sum }}r_{i}\left(t_{t-1}\right)-\lfloor \overset{n}{\underset{i=1}{\sum }}r_{i}\left(t_{t-1}\right)\rfloor \leq 1-r_{C\left(t_{t-1}\right)+1}\left(t_{t-1}\right)$$

(9) $$1-\left(\overset{n}{\underset{i=1}{\sum }}r_{i}\left(t_{t-1}\right)-\lfloor \overset{n}{\underset{i=1}{\sum }}r_{i}\left(t_{t-1}\right)\rfloor \right)\geq r_{C\left(t_{t-1}\right)+1}\left(t_{t-1}\right)$$

 
☐

**Lemma 1.**  *(Total local utilization at event-t) At $t_{t}$, an event-t occurs where $\sum_{i=1}^{n}r_{i,t_{t}}=C\left(t_{t-1}\right)-1$, only if $\sum_{i=1}^{n}r_{i}\left(t_{t-1}\right)<C\left(t_{t-1}\right)$*.

**Proof.**  When an event-t occurs at $t_{t}$, the time interval between event-t and the immediately previous event, $t_{t}-t_{t-1}$, is computed as shown below:

(10) $$t_{t}-t_{t-1}=\left(t_{f}-t_{t-1}\right)-\left(C\left(\;t_{t-1}\right)-\overset{n}{\underset{i=1}{\sum }}r_{i}\left(t_{t-1}\right)\right)\left(t_{f}-t_{t-1}\right)\;=\left(t_{f}-t_{t-1}\right)\left(1-\left(C\left(\;t_{t-1}\right)-\overset{n}{\underset{i=1}{\sum }}r_{i}\left(t_{t-1}\right)\right)\right)$$

At $t_{t-1}$, the total local remaining execution time is computed as follows:

(11) $$\overset{n}{\underset{i=1}{\sum }}l_{i,t-1}=\left(t_{f}-t_{t-1}\right)\left(\overset{n}{\underset{i=1}{\sum }}r_{i}\left(t_{t-1}\right)\right)$$

From $t_{t-1}$ to $t_{t}$, the total local remaining execution time is reduced by as much as $C\left(t_{t-1}\right)\left(t_{t}-t_{t-1}\right)$.

(12) $$\begin{array}{ll} \left(t_{f}-t_{t}\right)\sum_{i=1}^{n}r_{i}\left(t_{t}\right) & \\ & =\left(t_{f}-t_{t-1}\right)\left(\sum_{i=1}^{n}r_{i}\left(t_{t-1}\right)\right)\\ & -C\left(t_{t-1}\right)\left(t_{f}-t_{t-1}\right)\left(1-\left(C\left(\;t_{t-1}\right)-\sum_{i=1}^{n}r_{i}\left(t_{t-1}\right)\right)\right) \end{array}$$

Since $t_{f}-t_{t-1}/t_{f}-t_{t}=1/C\left(t_{t-1}\right)-\sum_{i=1}^{n}r_{i}\left(t_{t-1}\right)$, we can replace $t_{f}-t_{t-1}/t_{f}-t_{t}$ with $1/C\left(t_{t-1}\right)-\sum_{i=1}^{n}r_{i}\left(t_{t-1}\right)$ as follows:

(13) $$\overset{n}{\underset{i=1}{\sum }}r_{i}\left(t_{t}\right)=\frac{t_{f}-t_{t-1}}{t_{f}-t_{t}}\left(C\left(t_{t-1}\right)-1\right)\left(C\left(t_{t-1}\right)-\overset{n}{\underset{i=1}{\sum }}r_{i}\left(t_{t-1}\right)\right)$$

(14) $$\overset{n}{\underset{i=1}{\sum }}r_{i}\left(t_{t}\right)=\frac{1}{C\left(t_{t-1}\right)-\sum_{i=1}^{n}r_{i}\left(t_{t-1}\right)}\left(C\left(t_{t-1}\right)-1\right)\left(C\left(t_{t-1}\right)-\overset{n}{\underset{i=1}{\sum }}r_{i}\left(t_{t-1}\right)\right)$$

Hence, the following equation holds:

(15) $$\overset{n}{\underset{i=1}{\sum }}r_{i}\left(t_{t}\right)=C\left(t_{t-1}\right)-1$$

 
☐

**Lemma 2.**  *If $\sum_{i=1}^{n}r_{i}\left(t_{cur}\right)=C\left(t_{cur\;}\right)$ at $t_{cur}$, then the total local utilization is $\sum_{i=0}^{n}r_{i}\left(t_{cur}\right)$ constantly at any time point, $t_{cur}+\Delta t$, where Δt>0*.

**Proof.**  (16) $$\overset{n}{\underset{i=1}{\sum }}r_{i}\left(t_{cur}\right)=C\left(t_{cur}\right)$$

(17) $$\overset{n}{\underset{i=1}{\sum }}\frac{l_{i}\left(t_{cur}\right)}{t_{f}-t_{cur}}=C\left(t_{cur}\right)$$

(18) $$\overset{n}{\underset{i=1}{\sum }}l_{i}\left(t_{cur}\right)=C\left(t_{cur}\right)\left(t_{f}-t_{cur}\right)$$

(19) $$\overset{n}{\underset{i=1}{\sum }}l_{i}\left(t_{cur}\right)-\Delta tC\left(t_{cur}\right)=C\left(t_{cur}\right)\left(t_{f}-t_{cur}\right)-\Delta tC\left(t_{cur}\right)$$

(20) $$\overset{n}{\underset{i=1}{\sum }}l_{i}\left(t_{cur}+\Delta t\right)<C\left(t_{cur}\right)\left(t_{f}-t_{cur}-\Delta t\right)$$

(21) $$\frac{\sum_{i=1}^{n}l_{i}\left(t_{cur}+\Delta t\right)}{t_{f}-(t_{cur}+\Delta t)}=C\left(t_{cur}\right)$$

Hence, the following equation holds:

(22) $$\overset{n}{\underset{i=1}{\sum }}r_{i}\left(t_{cur}+\Delta t\right)=C\left(t_{cur}\right)$$

 
☐

**Theorem 2.**  *(The number of occurrences of event-t) Event-t occurs at most once in each plane*.

**Proof.**  Assume that an event-t occurs at time $t_{t}$ in a plane. Lemma 1 implies that $\sum_{i=1}^{n}r_{i}\left(t_{t}\right)=\;C\left(t_{t-1}\right)-1$, which means that only $C\left(t_{t-1}\right)-1$ processors are needed to handle the task load. Note that $C\left(t_{t}\right)=\sum_{i=1}^{n}r_{i}\left(t_{t}\right)$ at $t_{t}$ and the total local utilization is fixed to $C\left(t_{t-1}\right)-1$ between the times $t_{t}$ and $t_{f}$ by Lemma 2. Therefore, there cannot exist any other event-t in this plane. ☐

Within any T-L plane, the total local utilization of the tasks is bounded as shown in the following theorems.

**Theorem 3.**  If $\sum_{i=1}^{n}r_{i}\left(t_{cur}\right)<C\left(t_{cur}\right)$ at $t_{cur}$ and there is no idle time until $t_{cur}+\Delta t$ where Δt>0, then the total local utilization at $t_{cur}+\Delta t$ is smaller than $\sum_{i=1}^{n}r_{i}\left(t_{cur}\right)$, i.e., $\sum_{i=1}^{n}r_{i}\left(t_{cur}+\Delta t\right)<\sum_{i=1}^{n}r_{i}\left(t_{cur}\right)$.

**Proof.**  Since $\sum_{i=1}^{n}r_{i}\left(t_{cur}\right)<C\left(t_{cur}\right)$ at $t_{cur}$, the idle time after that is $C\left(t_{cur}\right)\left(t_{f}-t_{cur}\right)-\sum_{i=1}^{n}l_{i}\left(t_{cur}\right)$. Since there is no idle time during $(t_{cur},\;t_{cur}+\Delta t$), the idle time at $t_{cur}+\Delta t$ can be computed as shown in the following:

(23) $$\begin{array}{lll} idle_{t_{cur+\Delta t}} & = & C\left(t_{cur}\right)\left(t_{f}-t_{cur}-\Delta t\right)-\sum_{i=1}^{n}l_{i}\left(t_{cur}+\Delta t\right)\\ & = & C\left(t_{cur}\right)\left(t_{f}-t_{cur}\right)-\sum_{i=1}^{n}l_{i}\left(t_{cur}\right) \end{array}$$

The total local utilization at $t_{cur}$ is computed as shown below:

(24) $$\overset{n}{\underset{i=1}{\sum }}r_{i}\left(t_{cur}\right)=C\left(t_{cur}\right)-\frac{idle_{t_{cur}}}{\left(t_{f}-t_{cur}\right)}$$

The total local utilization at $t_{cur}+\Delta t$ is computed as follows:

(25) $$\overset{n}{\underset{i=1}{\sum }}r_{i}\left(t_{cur}+\Delta t\right)=C\left(t_{cur}\right)-\frac{idle_{t_{cur}}}{\left(t_{f}-t_{cur}-\Delta t\right)}$$

Since $\left(t_{f}-t_{cur}\right)>\left(t_{f}-t_{cur}-\Delta t\right)$ holds, the following is satisfied as well:

(26) $$\overset{n}{\underset{i=1}{\sum }}r_{i}\left(t_{cur}+\Delta t\right)<\overset{n}{\underset{i=1}{\sum }}r_{i}\left(t_{cur}\right)$$

 
☐

Algorithm 2 shows an algorithm to handle the event-t, which switches unnecessary processors to sleep mode.
**Algorithm 2** Mode Transition at Event-t
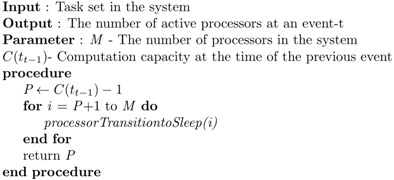


### 4.2. Prefetching Strategy

Kim et al. [7] proposed an event-s that executes the tokens originally scheduled on the next plane to reduce fragmented short idle intervals. However, the event-s was limited to handle the tokens on the immediate next plane only. In this paper, we propose to extend the event-s to consider all of the future planes. A naive attempt may increase the computation complexity from Θ(n) to $\Theta \left(n^{2}\right)$ because we need to calculate the local execution time of each token on n−1 planes constructed by n tasks. In order to solve this problem, we propose a new event called event-r. The occurrence of event-r requires the calculation of the remaining execution times of n tasks only. Thus, the algorithm complexity is maintained at Θ(n). In the remainder of this section, we describe the new event in detail.

**Definition 2.**  *An event-r occurs at $t_{r}$ if the following conditions are satisfied*.

- $t_{f}-t_{r}<C_{sleep}$
- $\sum_{i=1}^{n}r_{i}\left(t_{r}\right)<\sum_{i=1}^{n}r_{i}\left(t_{r-1}\right)$
- There exists no event-r during the time interval $\left(t_{f}-C_{sleep},\;t_{r}\right)$

**Figure 5:**
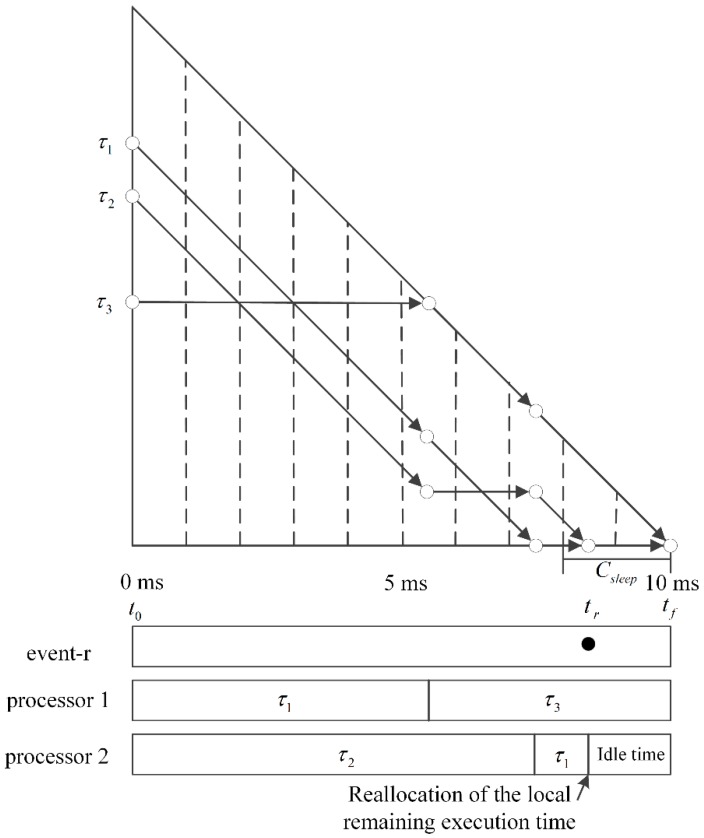
Example of the occurrence of an event-r.

Figure 5 shows an example of the T-L plane where an event-r occurs. From $t_{r}$ to $t_{f}$, the total processor time is $C\left(t_{r-1}\right)\left(t_{f}-t_{r}\right)$. At $t_{r}$, we reallocate the local remaining execution time to the future tokens originally scheduled to the next planes. The total local utilizations before and after reallocation at $t_{r}$ are denoted as $\sum_{i=1}^{n}r_{i}^{before}\left(t_{r}\right)$ and $\sum_{i=1}^{n}r_{i}^{after}\left(t_{r}\right)$, respectively. The processor time required by the tokens before reallocation is $\sum_{i=1}^{n}r_{i}^{before}\left(t_{r}\right)\left(t_{f}-t_{r}\right)$. Therefore, $\left(C\left(t_{r-1}\right)-\sum_{i=1}^{n}r_{i}^{before}\left(t_{r}\right)\right)\left(t_{f}-t_{r}\right)$ is distributed to the tokens in a plane. The additional processor time reallocated to the tokens is computed by the following theorems.

**Theorem 4.**  (Maximum additional processor time) The additional processor time $a_{i}$ to be reallocated to a token of task $\tau_{i}$ at event-r satisfies the inequality $a_{i}\leq \left(1-r_{i}^{before}\left(t_{r}\right)\right)\left(t_{f}-t_{r}\right)$.

**Proof.**  At $t_{r}$, the maximum available processor time by a task $\tau_{i}$ is $t_{f}-t_{r},$ and $l_{i}^{before}\left(t_{r}\right)$ is $r_{i}^{before}\left(t_{r}\right)\left(t_{f}-t_{r}\right)$.

(27) $$l_{i}^{after}\left(t_{r}\right)\leq t_{f}-t_{r}$$

(28) $$l_{i}^{before}\left(t_{r}\right)+a_{i}\leq t_{f}-t_{r}$$

(29) $$a_{i}\leq t_{f}-t_{r}-l_{i}^{before}\left(t_{r}\right)$$

Therefore, the following inequality holds for the additional processor time $a_{i}$:

(30) $$a_{i}\leq \left(1-r_{i}^{before}\left(t_{r}\right)\right)\left(t_{f}-t_{r}\right)$$

 
☐

**Theorem 5.**  (Total maximum additional processor time) The total additional processor time A reallocated to the tokens in a plane satisfies the inequality $A\leq \left(C\left(t_{r-1}\right)-\sum_{i=1}^{n}r_{i}^{before}\left(t_{r}\right)\right)\left(t_{f}-t_{r}\right).$

**Proof.**  (31) $$\sum_{i=1}^{n}r_{i}^{after}\left(t_{r}\right)\leq C\left(t_{r-1}\right)$$

(32) $$\sum_{i=1}^{n}r_{i}^{after}\left(t_{r}\right)-\sum_{i=1}^{n}r_{i}^{before}\left(t_{r}\right)\leq C\left(t_{r-1}\right)-\sum_{i=1}^{n}r_{i}^{before}\left(t_{r}\right)$$

(33) $$\sum_{i=1}^{n}\left(l_{i}^{after}\left(t_{r}\right)-l_{i}^{before}\left(t_{r}\right)\right)\leq \left(C\left(t_{r-1}\right)-\sum_{i=1}^{n}r_{i}^{before}\left(t_{r}\right)\right)\left(t_{f}-t_{r}\right)$$

Hence, the following inequality is obtained:

(34) $$A\leq \left(C\left(t_{r-1}\right)-\overset{n}{\underset{i=1}{\sum }}r_{i}\left(t_{r}\right)\right)\left(t_{f}-t_{r}\right)$$

 
☐

The following theorem presents a sufficient condition for the occurrence of an event-r in a plane.

**Theorem 6.**  *There exists no event-r in a plane if an event-t occurs in the plane*.

**Proof.**  An event-t occurs at t where $t_{0}<t\leq t_{f}-C_{sleep}$. Also, an event-r occurs at t where $t_{f}-C_{sleep}<t<t_{f}$. Therefore, we will prove that there cannot be any event-r after the occurrence of an event-t. Suppose that an event-r occurs in the plane after the occurrence of an event-t. When an event-t occurs at $t_{t}$, the total local utilization is $\sum_{i=1}^{n}r_{i,t_{t}}=C\left(t_{t-1}\right)-1$ according to Lemma 1 and the computation capacity required for scheduling tasks is computed as $C\left(t_{t}\right)=C\left(t_{t-1}\right)-1$. Since the total local utilization is $\sum_{i=1}^{n}r_{i}\left(t_{t+\Delta t}\right)=\sum_{i=1}^{n}r_{i}\left(t_{t}\right)$ at $t_{t}+\Delta t$ where Δt>0, according to Lemma 2, the second condition of Definition 2 cannot be satisfied, which implies a contradiction. ☐

Algorithm 3 presents the algorithm description for the processor time reallocation at the occurrence of an event-r.
**Algorithm 3** Processor Time Reallocation at Event-r
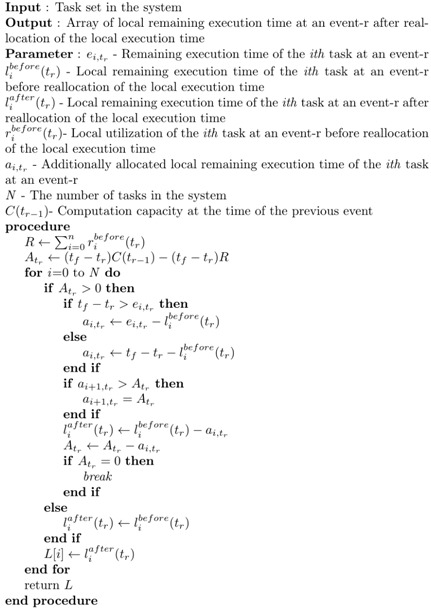


## 5. Experimental Results and Analysis

In this section, we compare the performance of the proposed algorithm with major real-time scheduling algorithms developed for efficient power management. For the experiments, we implemented a simulator using the Ruby language in Windows. The simulator can calculate energy consumption overheads associated with the state transitions for each scheduling algorithm, as well as the consumption for task executions. The experimental parameters of the simulator are set to reflect the characteristics of Marvell’s XScale-based processor PXA270 [30], which supports six voltage-frequency levels and five processor modes, as shown in Table 1 and Table 2. This particular processor is adopted in a wireless multimedia sensor network platform called CITRIC [31] and was used in recent studies [32,33]. It is anticipated that more processors for high-end embedded systems will be equipped with VFS and DPM [6,11].

To measure the scalability of the algorithms, we varied the number of available processors from 4–32. For each trial, we generated 100 task sets of which the utilization is fixed to four, and the rate of each task was varied in the range of [0.01, 0.99] by following the Emberson procedure [34]. Each task period is uniformly distributed over a range of [15, 150] and simulations were run for 1000 system time units.

The experiment includes the algorithms proposed by Funaoka et al. [24], which are referred to as uniform RT-SVFS and independent RT-SVFS. They are known to be state-of-the-art SVFS-based scheduling algorithms for both uniform and independent multiprocessors. The experiment also includes earlier approaches to DPM enabled T-L plane-based scheduling algorithms, such as TL-DPM [7] and LNREF with DPM [7]. As a baseline, the original LNREF was considered as well. We implemented the models for these algorithms on the simulator, as well as the proposed algorithm. Table 3 summarizes the characteristics of the algorithms. In case all the multiprocessors of a platform are assumed to have the same characteristics, the platform is referred to as “identical” type. A “uniform” type platform allows the processors at different speeds, but they are identical otherwise. Every job receives the same speed-up when assigned to faster processors. An “independent” type platform has independent computing characteristics on every processor. A job may experience different speed-up when assigned to different processors. Such a platform is also referred to as “unrelated”.

All of the T-L plane abstraction based scheduling algorithms discussed here are global optimal ones. Hence, there is no deadline miss as long as the total utilization is under the system capacity. The computational complexity of every algorithm under discussion is Θ(nlogn) due to the burden of sorting tasks in the order of local remaining execution time.

**Figure 6:**
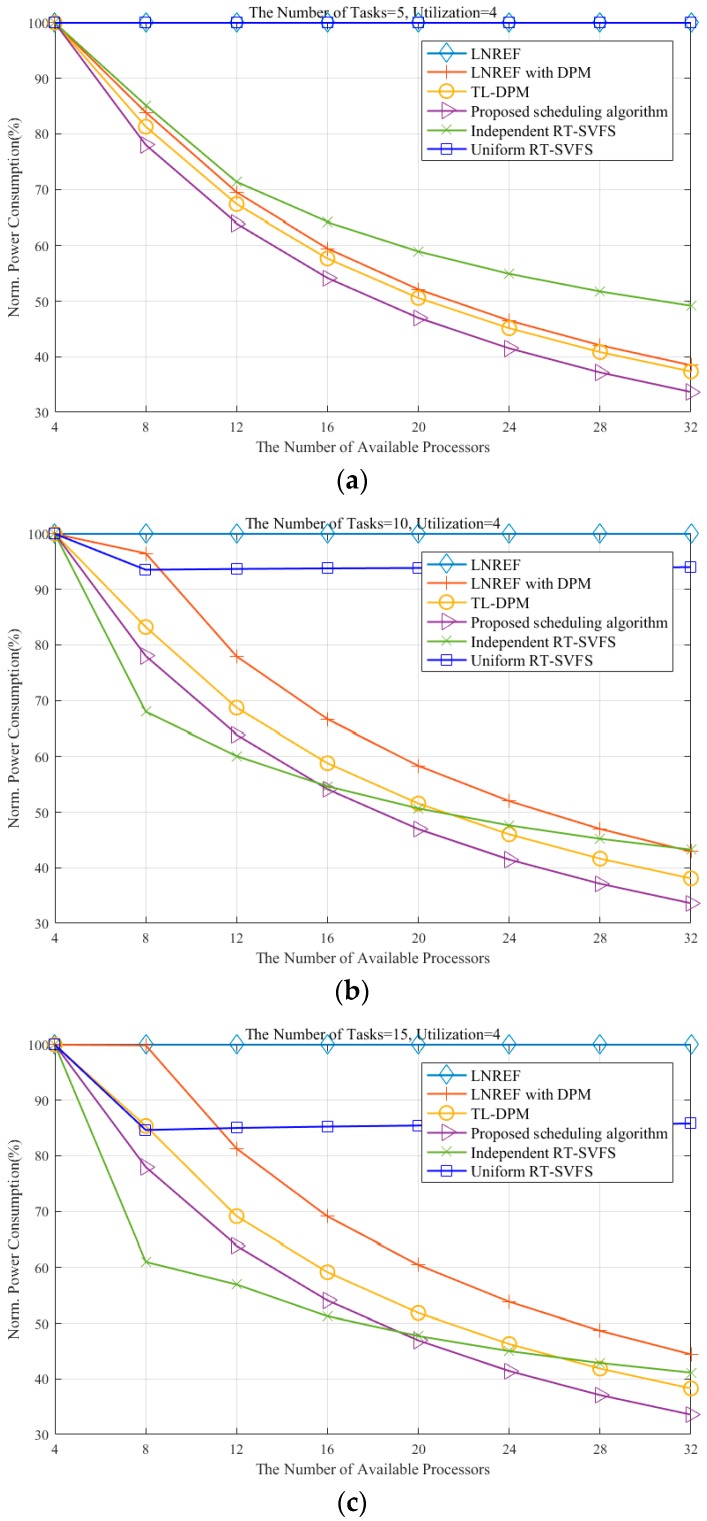

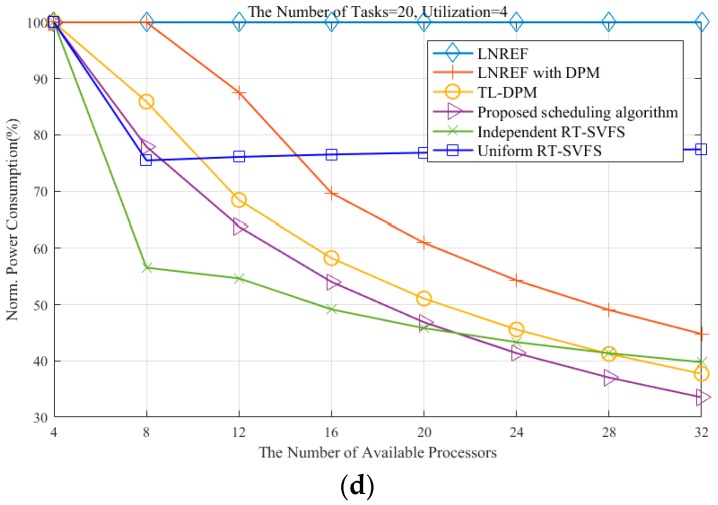
Comparison of energy-efficient approaches for T-L plane abstraction. The number of tasks is (**a**) 5, (**b**) 10, (**c**) 15, and (**d**) 20.

Figure 6 shows the power consumption measures for the six algorithms mentioned above: LNREF—the original LLREF algorithm without any power management; LNREF with DPM—a trivial extension to LNREF for DPM [7]; TL-DPM—a recent extension to T-L plane-based scheduling [7]; our proposed scheduling algorithm, independent RT-SVFS [24]; and uniform RT-SVFS [24]. The X-axis shows the number of available processors and the Y-axis represents the normalized power consumption (NPC), which is the ratio of the power consumption of an algorithm to that of LLREF. The task set sizes indicating the number of tasks in the task set are set to 5, 10, 15, and 20 in (a), (b), (c), and (d), respectively.

Notice that the NPC of every algorithm reaches 100% when the number of available processors is four because we intentionally adjusted the total utilization of each task set to four. It is observed that the algorithms utilizing the DPM technique exhibit better performance when the size of the task set (the number of tasks in the task set) is small and the task utilization is high, as shown in Figure 6a. In contrast, the algorithms based on the RT-SVFS technique show better performance when the task set size is large and the task utilization is low, as shown in Figure 6d.

Notice that the uniform RT-SVFS guarantees meeting the deadlines of a task set with the total utilization being less than or equal to αM and the maximum utilization of tasks being less than or equal to α on M processors with a frequency of α. When the number of processors in the simulation is increased from 8 to 32, the uniform RT-SVFS allows the processors to run with a frequency equal to the maximum utilization of tasks. Therefore, the results from the uniform RT-SVFS are shown to be constant even when there are more than eight processors, as shown in Figure 6b–d.

When scheduling a task set of which the total utilization is less than or equal to αM on M processors running at a frequency of α, the dependent RT-SVFS algorithm classifies the tasks triggering deadline misses into the heavy task set and allocates a dedicated processor to each heavy task exclusively. Therefore, the NPC of the dependent RT-SVFS algorithm plummets until there are eight available processors, as shown in Figure 6b–d. The NPC of dependent RT-SVFS monotonically decreases as the number of available processors increases in all cases, unlike uniform RT-SVFS.

Since the LNREF with DPM approach produces schedules by utilizing all available processors, even when not all of them are needed, increasing the number of tasks renders more fragmentations of the idle time in general. This behavior was also confirmed in our experiments, where the NPC of the LNREF with DPM approach increases as the number of tasks was increased, as shown in Figure 6. In order to reduce fragmentation of the idle time, the TL-DPM algorithm steals the local execution time of tokens originally scheduled to the next plane, which helps to prevent frequent occurrences of idle time whose duration is not long enough to switch to sleep mode. We observe that TL-DPM consumes less power compared to the LNREF with DPM approach, as shown in Figure 6.

Our proposed algorithm consumes the minimum number of processors needed to schedule a task set and reallocates the local remaining execution time incurred by idle durations that are not long enough to switch the processor to sleep mode. The experimental results show that the proposed algorithm consistently provides better power management in every case compared to the LNREF with DPM and TL-DPM, as shown in Figure 6. It should be noted that when the number of available processors is large enough for the task load, our proposed algorithm outperforms the independent RT-SVFS. We suspect that this is due to the limitations of the independent RT-SVFS, as it does not consider the tokens scheduled to the future planes and wastes energy by letting unassigned processors remain idle instead of switching them to sleep mode.

In addition, the proposed algorithm exhibits consistent performance with respect to the number of tasks, whereas the performance of the VFS-based approaches fluctuates with different task sets. More specifically, if each task set requires the same utilization, the proposed algorithm shows the same performance regardless of the other characteristics of the task sets, as shown in Figure 6. This is because the behavior of our proposed algorithm is mainly controlled by the total utilization of the task set. For example, if the proposed algorithm is given two task sets requiring the same utilization, then the two schedules produced by the algorithm have exactly the same mode transitions (i.e., active, idle, sleep, and deep sleep) during the same durations.

Table 4 and Table 5 summarize the main results of the experiments. Table 4 shows that the percentage of power consumption saving from our proposed algorithm remained stable when the number of tasks are varied. This is due to the performance of the proposed algorithm is not affected by the number of tasks, but only by the total utilization. It is also notable that SVFS algorithms perform better when the number of tasks is high; however, our proposed algorithm outperforms them when the number of tasks are low. Table 5 clearly shows the advantage of the proposed algorithm. It can cope with the increased computing power and exploits the maximum energy saving among the T-L plane based algorithms.

## 6. Conclusions and Future Work

There has been little work in the area of energy-efficient scheduling on T-L plane abstractions. In this paper, we present a new T-L plane-based scheduling algorithm for DPM-enabled multi-processors, which considers mode transition overhead and reduces fragmentations of the idle time. The issue of fragmentations of the idle time is inherent in T-L plane-based algorithms and we solve this problem by introducing three new events: the arrival event, event-t (transition event), and event-r (reallocation event). We implemented a simulator to measure the power consumption of various scheduling algorithms. The experimental results show that the proposed algorithm consistently outperforms other DPM-based approaches for T-L plane abstraction. In addition, the proposed algorithm provides better scalability to the number of available processors than VFS-based approaches.

Currently, our proposed algorithm can handle periodic tasks with implicit deadlines. In future work, we plan to extend our approach to handle sporadic tasks with constrained deadlines as well. It would also be very interesting to combine VFS and DPM approaches for T-L plane abstraction. We are planning to extend our experiments on actual platforms. In addition, the studies on trade-offs between power usage and the computational complexity, as well as performance evaluations on overloaded situations, are interesting, potential future studies.

## Figures and Tables

**Figure 2:**
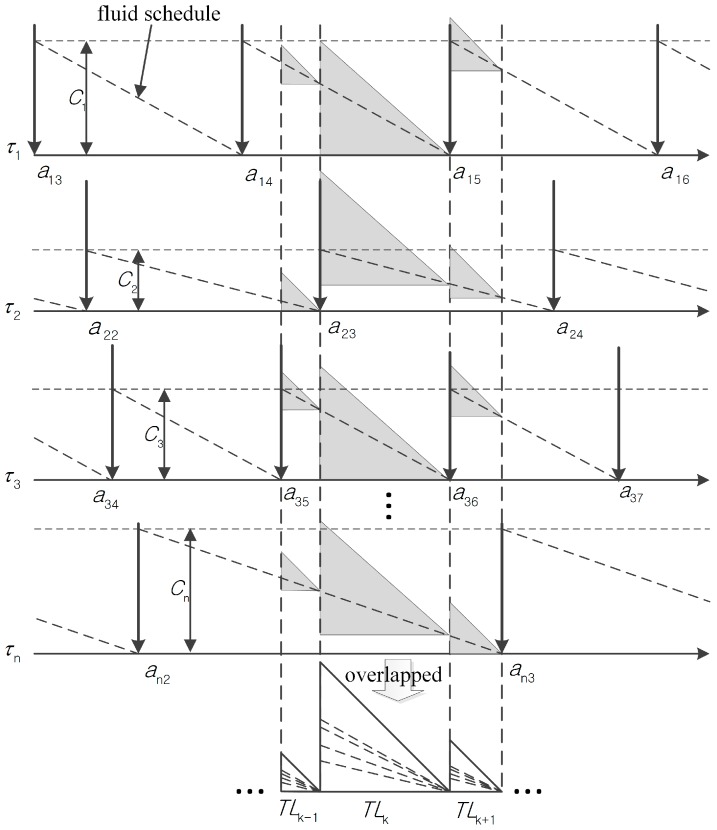
Example of T-L plane construction.

**Figure 3:**
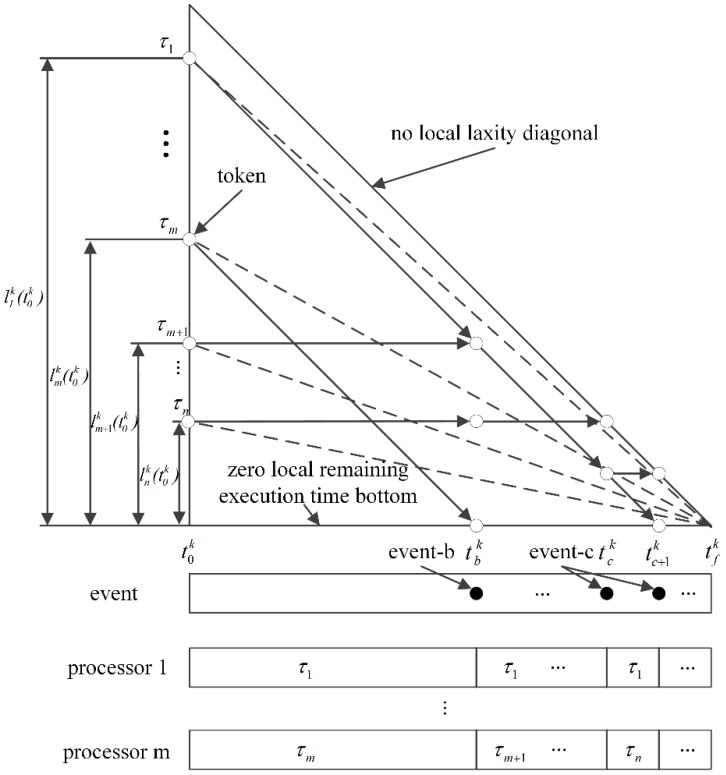
Scheduling in the *k-th* T-L plane.

**Table 1:** Voltage-frequency levels of the PXA270 processor [30].

Parameter	Level1	Level2	Level3	Level4	Level5	Level6
Frequency (MHz)	624	520	416	312	208	104
Active Power (mWatts)	925	675	468	301	279	116
Idle Power (mWatts)	260	222	186	154	129	64

**Table 2:** Power states of the PXA270 processor [30].

States	Power (mWatts)	Recovery Time (ms)
Running	925	0
Idle	260	0.001
Standby	1.722	11.43
Sleep	0.163	136.65
Deep sleep	0.101	261.77

**Table 3:** Summary of T-L plane based scheduling algorithms.

Algorithm Name	Platform Type	Power Management
LNREF	Identical	-
LNREF with DPM	Identical	DPM
TL-DPM	Identical	DPM
Proposed scheduling algorithm	Identical	DPM
Independent RT-SVFS	Independent	SVFS
Uniform RT-SVFS	Uniform	SVFS

**Table 4:** Summary of experimental results on varying number of tasks.

# of Processors	# of Tasks	Saved Norm. Power Consumption (%)				
	(total utilization)	LLREF with DPM	TL-DPM	Proposed algorithm	Independent RT-SVFS	Uniform RT-SVFS
8	5(4)	16	19	22	15	0
8	10(4)	4	17	22	32	6
8	15(4)	0	14	22	39	15
8	20(4)	0	14	22	43	24

**Table 5:** Summary of experimental results on varying number of processors.

# of Processors	# of Tasks	Saved Norm. Power Consumption (%)				
	(total utilization)	LLREF with DPM	TL-DPM	Proposed algorithm	Independent RT-SVFS	Uniform RT-SVFS
8	20(4)	0	14	22	43	24
12	20(4)	13	32	36	45	23
16	20(4)	30	42	46	51	23
20	20(4)	39	49	54	55	13
24	20(4)	46	55	59	57	13
28	20(4)	51	59	63	59	13
32	20(4)	55	62	66	60	13
